# Supplementary material for: BioGraphX: bridging the sequence-structure gap via physicochemical graph encoding for interpretable subcellular localization prediction
Source: Bioinform Adv. 2026 Jun 23;6(1):vbag181. doi: 10.1093/bioadv/vbag181 (PMC13355596; doi:10.1093/bioadv/vbag181)
Supplement: vbag181_Supplementary_Data [file vbag181_supplementary_data.pdf]

Supplementary Materials for:
BioGraphX: Bridging the Sequence-Structure Gap via Physicochemical Graph Encoding for Interpretable Subcellular Localization Prediction

Abubakar Saeed[1], Waseem Abbas[2,*]

[1]Department of Computer Science, Government College University Faisalabad, Chiniot Campus
[2]Corresponding author: waseem.abbas@gcuf.edu.pk

Supplementary Material S1: Complete BioGraphX Feature Descriptions (157 Features)

This supplementary document provides a comprehensive description of all 157 features extracted by the BioGraphX framework. The features are derived from a biochemically-calibrated constraint graph constructed directly from protein sequences. The framework's multi-scale approach captures protein topology, interaction networks, physicochemical properties, and constraint frustration to create a structural proxy without requiring 3D coordinates Gligorijević et al. (2021).

0.1 1. Topological Graph Features (85 features)

Description: Network architecture metrics derived from the biochemical interaction graph, applying concepts from graph theory to model protein residue relationships.

Table S1: Topological Graph Features (85 total)

Feature Name	Description	Biological Interpretation / Basis
Node_Count	Number of residues (vertices) in the graph.	Protein length.
Edge_Count	Number of biochemical interactions (edges) in the graph.	Overall connectivity.
Graph_Density	Ratio of actual edges to possible edges.	Compactness of interaction network.
Mean_Degree	Average number of interactions per residue.	Average residue connectivity.
Degree_Std	Standard deviation of degree distribution.	Heterogeneity in local connectivity.
Max_Degree	Maximum number of interactions for any residue.	Identification of highly connected hubs.
Degree_Q1	First quartile of degree distribution.	Lower bound of connectivity.
Degree_Median	Median of degree distribution.	Central tendency of connectivity.
Degree_Q3	Third quartile of degree distribution.	Upper bound of connectivity.
Mean_Weighted_Degree	Average edge-weighted degree (by strength).	Strength of interactions per residue.
Weighted_Degree_Std	Variability in weighted degree.	Diversity in interaction strengths.
Max_Weighted_Degree	Maximum weighted degree.	Residue with strongest total interactions.
Hydrophobic_Ratio	Proportion of hydrophobic interaction edges.	Dominance of hydrophobic packing.

Feature Name	Description	Biological Interpretation / Basis
HydrogenBond_Ratio	Proportion of hydrogen bond edges.	Importance of polar interactions.
SaltBridge_Ratio	Proportion of salt bridge edges.	Electrostatic stabilization strength.
Pi_Interaction_Ratio	Proportion of π-interaction edges.	Aromatic stacking interactions.
Disulfide_Ratio	Proportion of disulfide bond edges.	Extracellular/oxidized environment.
CationPi_Ratio	Proportion of cation-πinteraction edges.	Charged-aromatic interactions.
VanDerWaals_Ratio	Proportion of van der Waals interaction edges.	Close-packing interactions.
CH_Pi_Ratio	Proportion of CH-πinteraction edges.	Weak hydrophobic-aromatic interactions Nishio et al. (2014).
NH_Pi_Ratio	Proportion of NH-πinteraction edges.	Polar-aromatic interactions.
CarbonylCarbonyl_Ratio	Proportion of carbonyl-carbonyl edges.	Backbone-backbone hydrogen bonding.
SulfurPi_Ratio	Proportion of sulfur-πedges.	Methionine/aromatic interactions Valley et al. (2012).
Backbone_Ratio	Proportion of backbone peptide bonds.	Chain connectivity and flexibility.
Mean_Betweenness	Average betweenness centrality.	Importance in information flow Brandes (2001).
Betweenness_Std	Standard deviation of betweenness.	Heterogeneity in pathway importance.
Max_Betweenness	Maximum betweenness centrality.	Critical bottleneck residues.
Min_Betweenness	Minimum betweenness centrality.	Peripheral residues.
Betweenness_Q1	First quartile of betweenness.	Lower bound of centrality.
Betweenness_Q3	Third quartile of betweenness.	Upper bound of centrality.
Mean_Closeness	Average closeness centrality.	Average proximity to all residues.
Closeness_Std	Standard deviation of closeness.	Heterogeneity in proximity.
Max_Closeness	Maximum closeness centrality.	Centrally located residues.
Min_Closeness	Minimum closeness centrality.	Remotely located residues.
Mean_Eigenvector	Average eigenvector centrality.	Influence in network neighborhoods.
Eigenvector_Std	Standard deviation of eigenvector.	Heterogeneity in neighborhood influence.
Community_Count	Number of detected communities.	Domain/module organization.
Modularity	Quality of community partition.	Strength of domain separation.
Mean_Community_Size	Average community size.	Typical domain size.
Community_Size_Std	Standard deviation of community sizes.	Heterogeneity in domain sizes.
Max_Community_Size	Largest community size.	Major structural domain.
Min_Community_Size	Smallest community size.	Minor structural element.
IntraCommunity_Edge_Ratio	Ratio of edges within communities.	Internal domain connectivity.
InterCommunity_Edge_Ratio	Ratio of edges between communities.	Inter-domain connectivity.
N_Term_Hydrophobic_Density	Hydrophobic density in N-terminal (1-30).	Signal peptide h-region indicator.
N_Term_Basic_Centrality	Centrality of basic residues in N-terminal.	Targeting signal importance.
N_Term_Clustering	Clustering coefficient in N-terminal.	Local compactness near start.
N_Term_Edge_Density	Edge density in N-terminal region.	Structural compactness near start.
C_Term_Surface_Exposure	Average degree in C-terminal (last 10).	Surface accessibility at terminus.
C_Term_Charged_Connectivity	Connectivity between charged in C-terminal.	Retention signal interactions.
C_Term_Terminal_Degree	Degree of the very last residue.	Terminal residue exposure.
Basic_Residue_Connectivity	Connectivity among basic (K,R,H) residues.	NLS cluster formation.
Basic_Cluster_Centrality	Centrality of basic residue clusters.	Importance of basic regions.
Basic_Cluster_Count	Number of connected basic clusters.	Multiple basic regions.

Feature Name	Description	Biological Interpretation / Basis
Max_Basic_Cluster_Size	Size of largest basic cluster.	Major basic region.
Hydrophobic_Cluster_ Connectivity	Connectivity among hydrophobic residues.	Hydrophobic core formation.
Hydrophobic_External_Edges	Edges from hydrophobic to non-hydrophobic.	Surface exposure of hydrophobic.
Hydrophobic_Cluster_Count	Number of hydrophobic clusters.	Multiple hydrophobic patches.
Max_Hydrophobic_Cluster	Size of largest hydrophobic cluster.	Major hydrophobic core.
Mean_Hydrophobic_Cluster_ Size	Average hydrophobic cluster size.	Typical hydrophobic patch size.
N_Term_Basic_Density	Basic residue density in N-terminal.	Mitochondrial/chloroplast targeting.
Basic_Gradient	Difference in basic density N- vs C-terminal.	Charge asymmetry.
Acidic_Gradient	Difference in acidic density N- vs C-terminal.	Acidic residue distribution.
Net_Charge_Density	Net charge per residue (KRH minus DE).	Overall electrostatic character.
Avg_Shortest_Path	Average shortest path length (LCC).	Global communication efficiency.
Graph_Diameter	Longest shortest path (LCC).	Maximum communication distance.
Global_Efficiency	Inverse of average shortest path length.	Network communication efficiency.
Local_Efficiency	Average efficiency of local neighborhoods.	Local communication robustness Brandes (2001).
Path_Length_Std	Standard deviation of sampled paths.	Heterogeneity in distances.
Max_Path_Length	Maximum sampled shortest path.	Extreme communication distance.
Clustering_Coefficient	Average local clustering coefficient.	Local cohesiveness.
Assortativity	Degree correlation coefficient.	Homophily in connectivity.
Graph_Radius	Minimum eccentricity.	Compactness measure.
Triangle_Count	Number of 3-node cliques.	Local motif density.
N_Region_Density	Edge density in first 30 residues.	N-terminal structural compactness.
M_Region_Density	Edge density in middle region.	Internal structural compactness.
C_Region_Density	Edge density in last 30 residues.	C-terminal structural compactness.
Mean_Edge_Weight	Average edge weight.	Average interaction strength.
Edge_Weight_Std	Standard deviation of edge weights.	Heterogeneity in strengths.
Max_Edge_Weight	Maximum edge weight.	Strongest individual interaction.
Min_Edge_Weight	Minimum edge weight.	Weakest individual interaction.
Edge_Weight_Q1	First quartile of edge weights.	Lower bound of strengths.
Edge_Weight_Median	Median edge weight.	Typical interaction strength.
Edge_Weight_Q3	Third quartile of edge weights.	Upper bound of strengths.
Edge_Weight_Range	Difference between max and min weights.	Span of interaction strengths.

0.2 2. Hybrid Interaction Features (22 features)

Description: Co-occurrence patterns of biochemical interactions where two interaction types occur simultaneously between the same residue pair.

Table S2: Hybrid Interaction Features (23 total)

Feature Name	Description	Biological Interpretation
SaltBridge_HBond_Hybrid	Frequency of salt bridge + hydrogen bond.	Enzyme active sites, charged polar motifs.
Hydrophobic_Pi_Hybrid	Frequency of hydrophobic + π-interaction.	Membrane protein anchoring.

Feature Name	Description	Biological Interpretation
CationPi_HBond_Hybrid	Frequency of cation-π+ hydrogen bond.	Nuclear localization signals.
Hydrophobic_VDW_Hybrid	Frequency of hydrophobic + van der Waals.	Hydrophobic core packing.
Pi_Cation_HBond_Hybrid	Frequency of π-interaction + hydrogen bond.	DNA/RNA binding interfaces.
CH_Pi_Hydrophobic_Hybrid	Frequency of CH-π+ hydrophobic.	Lipid bilayer interactions.
Sulfur_Aromatic_Hybrid	Frequency of sulfur-π+ aromatic.	Redox-active sites.
Carbonyl_Charge_Hybrid	Frequency of carbonyl-carbonyl + salt bridge.	Electrostatic clusters, active sites.
N_Terminal_Hybrid_Density	Hybrid density in N-terminal region (1-30).	Complex targeting signals.
C_Terminal_Hybrid_Density	Hybrid density in C-terminal region (last 10).	Complex retention signals.
Hydrophobic_Region_Hybrid_Density	Hybrid density in hydrophobic clusters.	Specialized hydrophobic motifs.
Basic_Region_Hybrid_Density	Hybrid density in basic residue clusters.	Specialized nuclear targeting.
Hybrid_Edge_Ratio	Proportion of edges that are hybrid.	Overall interaction complexity.
Hybrid_Subgraph_Density	Density of subgraph of hybrid edges.	Compactness of hybrid clusters.
Hybrid_Network_Path_Length	Average path in hybrid subgraph.	Efficiency of hybrid network.
Hybrid_Cluster_Count	Connected components in hybrid subgraph.	Modularity of hybrid interactions.
Hybrid_Cooccurrence_1	Highest co-occurrence score.	Most synergistic interaction pair.
Hybrid_Cooccurrence_2	Second highest co-occurrence score.	Secondary synergistic pair.
Hybrid_Cooccurrence_3	Third highest co-occurrence score.	Tertiary synergistic pair.
Hybrid_Diversity	Shannon entropy of hybrid types.	Diversity of hybrid interactions.
Hybrid_Network_Centrality	Average betweenness in hybrid subgraph.	Importance in hybrid network.
Hybrid_Clustering	Clustering coefficient of hybrid subgraph.	Local cohesiveness of hybrids.

0.3 3. Targeting Signal Features (20 features)

Description: Sequence-based localization signals derived from known biological motifs and hybrid scores, informed by databases of experimentally validated targeting signals Negi et al. (2015); Nielsen (2017).

Table S3: Knowledge-Guided Signal Features (20 total)

Feature Name	Description	Biological Interpretation
Signal_Nucleus_Residue	Residue-based NLS motif score.	Presence of monopartite/bipartite NLS.
Signal_Nucleus_Hybrid	Hybrid-based nuclear compatibility.	Structural compatibility with nucleus.
Signal_Mitochondrion_Residue	Residue-based MTS score.	Amphipathic helix and charge patterns.
Signal_Mitochondrion_Hybrid	Hybrid-based mitochondrial compatibility.	Compatibility with mitochondrial import.
Signal_Extracellular_Residue	Signal peptide grammar score.	n-region, h-region, c-region patterns.
Signal_Extracellular_Hybrid	Hybrid-based extracellular compatibility.	Structural compatibility with secretion.
Signal_Cell_membrane_Residue	Transmembrane domain score.	Hydrophobic runs for membrane insertion.
Signal_Cell_membrane_Hybrid	Hybrid-based membrane compatibility.	Compatibility with membrane environment.

Feature Name	Description	Biological Interpretation
Signal_Endoplasmic_reticulum_Residue	ER retention signal score.	KDEL/HDEL/KKXX motifs.
Signal_Endoplasmic_reticulum_Hybrid	Hybrid-based ER compatibility.	Compatibility with ER lumen/membrane.
Signal_Golgi_apparatus_Residue	Golgi targeting score.	Tyrosine motifs (YXXΦ), TMD properties.
Signal_Golgi_apparatus_Hybrid	Hybrid-based Golgi compatibility.	Structural compatibility with Golgi.
Signal_Lysosome_Vacuole_Residue	Lysosomal targeting score.	Dileucine motifs, glycosylation signals.
Signal_Lysosome_Vacuole_Hybrid	Hybrid-based lysosomal compatibility.	Compatibility with lysosomal environment.
Signal_Peroxisomal_Residue	Peroxisomal targeting score.	PTS1 (SKL) and PTS2 motifs.
Signal_Peroxisomal_Hybrid	Hybrid-based peroxisomal compatibility.	Compatibility with peroxisomal import.
Signal_Plastid_Residue	Plastid transit peptide score.	Ser/Thr-rich, low acidic N-terminal.
Signal_Plastid_Hybrid	Hybrid-based plastid compatibility.	Compatibility with chloroplast import.
Signal_Cytoplasm_Residue	Cytoplasmic default score.	Absence of strong targeting signals.
Signal_Cytoplasm_Hybrid	Hybrid-based cytoplasmic compatibility.	Compatibility with cytoplasmic environment.

0.4 4. Global Physicochemical Features (19 features)

Description: Whole-protein biophysical properties derived from amino acid composition and sequence Kyte and Doolittle (1982); Leuenberger et al. (2017), reflecting constraints important for protein evolution and stability Sikosek and Chan (2014); Rockah-Shmuel et al. (2015).

Table S4: Global Physicochemical Features (19 total)

Feature Name	Description	Biological Interpretation
Isoelectric_Point	Theoretical pI via bisection method.	Charge at physiological pH; solubility.
Net_Charge	Total charge at pH 7.0.	Overall electrostatic properties.
Charge_Density_Physics	Net charge per residue.	Charge distribution density.
GRAVY	Grand Average of Hydropathy (Kyte-Doolittle).	Hydrophobicity/solubility index Kyte and Doolittle (1982).
Aromaticity	Fraction of aromatic residues (F,Y,W).	Aromatic content affecting packing.
Instability_Proxy	Fraction of PEST/QD residues.	Proxy for protein stability Broom et al. (2017).
Hydro_Corr_L1	Hydrophobicity autocorrelation lag 1.	Alternating hydrophobicity patterns.
Hydro_Corr_L2	Hydrophobicity autocorrelation lag 2.	Periodic hydrophobicity (β-strands).
Hydro_Corr_L3	Hydrophobicity autocorrelation lag 3.	Periodic patterns.
Hydro_Corr_L4	Hydrophobicity autocorrelation lag 4.	α-helix periodicity.
Hydro_Corr_L5	Hydrophobicity autocorrelation lag 5.	Extended patterns.
Hydro_Corr_L6	Hydrophobicity autocorrelation lag 6.	Long-range patterns.
Charge_Corr_L1	Charge autocorrelation lag 1.	Alternating charge patterns.
Charge_Corr_L2	Charge autocorrelation lag 2.	Periodic charge distribution.
Charge_Corr_L3	Charge autocorrelation lag 3.	Periodic patterns.
Charge_Corr_L4	Charge autocorrelation lag 4.	Helical charge periodicity.
Charge_Corr_L5	Charge autocorrelation lag 5.	Extended patterns.
Charge_Corr_L6	Charge autocorrelation lag 6.	Long-range patterns.
Shannon_Entropy	Sequence complexity (amino acid distribution).	Evolutionary conservation/variability Dill et al. (2007).

0.5 5. Constraint Frustration Features (11 features)

Description: Per-residue conflicting interaction energies derived from variance in constraint graph weights. These features capture biophysical conflicts that serve as structural compatibility filters for challenging compartments.

Table S5: Constraint Frustration Features (11 total)

Feature Name	Description	Biological Interpretation
Frustration_NTerminal_Mean	Mean frustration in N-terminal region (residues 1-30).	Signal peptide vs. structural constraints.
Frustration_CTerminal_Mean	Mean frustration in C-terminal region (last 10 residues).	Retention signal vs. structural constraints.
Frustration_Structural_Mean	Mean frustration in middle structural region.	Core folding constraints.
Frustration_SignalVsStructure	Difference between N-terminal and structural frustration.	Signal region specificity indicator.
Frustration_MaxInSignalRegion	Maximum frustration value in N-terminal region.	Local constraint conflict hotspots.
Frustration_Localization	Inverse entropy of frustration distribution.	How localized vs. distributed conflicts are.
Frustration_HotspotCount	Number of residues with frustration ¿ mean + 2σ.	Count of severe constraint conflicts.
Frustration_SatisfactionRatio	Proportion of residues with frustration ¡ median.	Fraction of residues with compatible constraints.
Frustration_SignalCorrelation	Correlation between frustration and sequence Signal scores.	Conservation vs. constraint conflicts alignment.
Frustration_HighSignal Frustration	Binary flag for high frustration in signal region.	Indicates significant signal-region conflicts.
Frustration_PerResidue_Mean	Mean of per-residue frustration vector (first 100 residues).	Summary of overall constraint conflicts.

Supplementary Material S2: Feature Summary and SHAP Framework

Table S6 provides a summary of the five feature categories. The SHAP (SHapley Additive exPlanations) framework Lundberg and Lee (2017) was used to explain model predictions, providing theoretically sound quantification of each feature's contribution.

Table S6: Summary of BioGraphX Feature Categories (Total: 157 features)

Category	# Features	Scale	Biological Insight
Topological Graph	85	Residue to protein	Protein fold complexity, domain organization, network properties
Hybrid Interaction	22	Pairwise interactions	Specialized structural motifs, synergistic forces
Targeting Signal Features	20	Whole-protein	Known targeting signals + structural compatibility
Global Physicochemical	19	Whole-protein	Biophysical constraints, solubility, stability
Constraint Frustration	11	Per-residue	Conflicting interaction energies, structural compatibility filters
**Total**	**157**	**Multi-scale**	**Comprehensive sequence-structure-function representation**

Supplementary Material S3: Computational Details

The time complexity for feature extraction is $O(n^2)$ for graph construction (where n is sequence length), with optimizations for long sequences:

- **Short** ($\leq 2,000$ aa): Full processing
- **Medium** ($2,000 < L \leq 10,000$): Smart truncation (40% N, 20% middle, 40% C)
- **Long** ($> 10,000$ aa): Sliding window (window=1000, stride=500) with weighted aggregation

Supplementary Material S4: Parallel Implementation and Scalability

S4.1 Parallel Processing Architecture

The BioGraphX encoding pipeline employs a data-parallel architecture where independent protein sequences are processed concurrently. The implementation uses Python's Joblib library with the Loky backend for process-based parallelism. Key design decisions:

- **Embarrassingly parallel design**: Each protein sequence is encoded independently, enabling perfect scalability.
- **Dynamic batching**: Sequences are automatically grouped into batches sized according to available memory and core count.
- **Fault tolerance**: Worker processes are isolated to prevent single-sequence failures from affecting the entire batch.
- **Memory efficiency**: Large sequences (>10,000 aa) trigger adaptive processing before parallel distribution.

References

Brandes, U. (2001). A faster algorithm for betweenness centrality. *The Journal of Mathematical Sociology*, 25(2):163–177.

Broom, A., Jacobi, Z., Trainor, K., and Meiering, E. M. (2017). Computational tools help improve protein stability but with a solubility tradeoff. *The Journal of Biological Chemistry*, 292(35):14349–14361.

Dill, K. A., Ozkan, S. B., Weikl, T. R., Chodera, J. D., and Voelz, V. A. (2007). The protein folding problem: when will it be solved? *Current Opinion in Structural Biology*, 17(3):342–346. Nucleic acids / Sequences and topology.

Gligorijević, V., Renfrew, P. D., Kosciolek, T., Leman, J. K., Berenberg, D., Vatanen, T., Chandler, C., Taylor, B., Fisk, I., Vlamakis, H., Xavier, R. J., Knight, R., Cho, K., and Bonneau, R. (2021). Structure-based protein function prediction using graph convolutional networks. *Nature Communications*, 12:3168.

Kyte, J. and Doolittle, R. F. (1982). A simple method for displaying the hydropathic character of a protein. *Journal of Molecular Biology*, 157(1):105–132.

Leuenberger, P., Ganscha, S., Kahraman, A., Cappelletti, V., Boersema, P. J., von Mering, C., Claassen, M., and Picotti, P. (2017). Cell-wide analysis of protein thermal unfolding reveals determinants of thermostability. *Science*, 355(6327):eaai7825.

Lundberg, S. M. and Lee, S.-I. (2017). A unified approach to interpreting model predictions. In *Proceedings of the 31st International Conference on Neural Information Processing Systems*, NIPS'17, page 4768–4777, Red Hook, NY, USA. Curran Associates Inc.

Negi, S., Pandey, S., Srinivasan, S. M., Mohammed, A., and Guda, C. (2015). Locsigdb: a database of protein localization signals. *Database*, 2015:bav003.

Nielsen, H. (2017). Predicting secretory proteins with signalp. In *Methods in Molecular Biology*, volume 1611, pages 59–73. Humana Press.

Nishio, M., Umezawa, Y., Fantini, J., Weiss, M. S., and Chakrabarti, P. (2014). Ch–π hydrogen bonds in biological macromolecules. *Phys. Chem. Chem. Phys.*, 16:12648–12683.

Rockah-Shmuel, L., Tóth-Petróczy, Á., and Tawfik, D. S. (2015). Systematic mapping of protein mutational space by prolonged drift reveals the deleterious effects of seemingly neutral mutations. *PLoS Computational Biology*, 11(8):e1004421.

Sikosek, T. and Chan, H. S. (2014). Biophysics of protein evolution and evolutionary protein biophysics. *Journal of The Royal Society Interface*, 11(100):20140419.

Valley, C. C., Cembran, A., Perlmutter, J. D., Lewis, A. K., Labello, N. P., Gao, J., and Sachs, J. N. (2012). The methionine–aromatic motif plays a unique role in stabilizing protein structure. *The Journal of Biological Chemistry*, 287(42):34979–34991.
